# Epigenetic Mechanism of Early Life Stress-Induced Depression: Focus on the Neurotransmitter Systems

**DOI:** 10.3389/fcell.2022.929732

**Published:** 2022-07-05

**Authors:** Ziqian Cheng, Jingyun Su, Kai Zhang, Huiyi Jiang, Bingjin Li

**Affiliations:** ^1^ Jilin Provincial Key Laboratory on Molecular and Chemical Genetic, Second Hospital of Jilin University, Changchun, China; ^2^ Engineering Lab on Screening of Antidepressant Drugs, Jilin Province Development and Reform Commission, Changchun, China; ^3^ Central Laboratory, The Second Hospital of Jilin University, Jilin University, Changchun, China; ^4^ Department of Pediatrics, The First Hospital of Jilin University, Changchun, China

**Keywords:** early life stress, depression, epigenetics, neurotransmitter systems, methylation

## Abstract

Depression has an alarmingly high prevalence worldwide. A growing body of evidence indicates that environmental factors significantly affect the neural development and function of the central nervous system and then induce psychiatric disorders. Early life stress (ELS) affects brain development and has been identified as a major cause of depression. It could promote susceptibility to stress in adulthood. Recent studies have found that ELS induces epigenetic changes that subsequently affect transcriptional rates of differentially expressed genes. The epigenetic modifications involved in ELS include histone modifications, DNA methylation, and non-coding RNA. Understanding of these genetic modifications may identify mechanisms that may lead to new interventions for the treatment of depression. Many reports indicate that different types of ELS induce epigenetic modifications of genes involved in the neurotransmitter systems, such as the dopaminergic system, the serotonergic system, the gamma-aminobutyric acid (GABA)-ergic system, and the glutamatergic system, which further regulate gene expression and ultimately induce depression-like behaviors. In this article, we review the effects of epigenetic modifications on the neurotransmitter systems in depression-like outcomes produced by different types of ELS in recent years, aiming to provide new therapeutic targets for patients who suffer from depression.

## Introduction

Early life stress (ELS) includes adverse gestational (maternal stress and maternal infection) and adverse childhood experiences (parental loss, emotional abuse, and physical abuse) (Oh et al., 2013; Provenzi et al., 2018). Early life is a critical time for axonal growth and synaptic growth, and in this period, the interaction with mothers is crucial. Studies have pointed out that offspring form special and strong emotional bonds with their caregivers in early life. When this attachment relationship is destroyed, it affects the offspring’s emotional, cognitive, and behavioral responsiveness (Sroufe, 2005; Feifel et al., 2017). When exposed to a long-term maternal separation, rat pups become increasingly marked with slower developing changes in behavioral reactivity, unresponsiveness, reduced movement, reduced alertness, food neglect, and indifference to new stimuli (Hofer, 1970, Hofer, 1973; Hofer, 1994). As Harlow indicated, one of the main roles of the mother is to shape the behavioral responsiveness of young monkeys and to guide the infant in age-appropriate social behavior with peers and adults (Harlow et al., 1971; Hall and Perona, 2012). When rhesus monkeys were exposed to maternal deprivation and then grew up to become mothers, they were extremely abusive and neglectful of their infants (Seay et al., 1964; Arling and Harlow, 1967). This pattern of abnormal behavior may be passed on from generation to generation, as maternal deprivation can lead to abnormal behaviors in offspring, resulting in abnormal behaviors in the next generation of mothers (Hall and Perona, 2012). It has been found that ELS increases susceptibility to psychiatric disorders in adulthood, including depression, anxiety, schizophrenia, autism, and attention deficit hyperactivity disorder (Gilbert et al., 2009; van Velzen and Toth, 2010; Deslauriers et al., 2013; Oh et al., 2013; Shepard and Nugent, 2020; Kronman et al., 2021). Depressed patients who suffered childhood trauma have faster brain aging and have a longer duration of illness than those who have not suffered childhood trauma (Klein et al., 2009; Miniati et al., 2010). In addition, the effect of ELS on depression-like behaviors is related to the duration of stress exposure. It has been reported that long-term maternal separation increases despair-like behaviors, while short-term maternal separation produces better adaptation to stress in adulthood (Köhler et al., 2019). The hypothalamic-pituitary-adrenal (HPA) axis is the primary stress response system. Cortisol mediates numerous tissue-specific effects through the glucocorticoid receptor (Young et al., 2003; Farrell et al., 2018). It has been found that early-life adversity leads to a lifelong increase in glucocorticoid secretion and disruption of the homeostasis of HPA axis activity, and a disturbance of the HPA axis will lead to vulnerability to depression (Heim and Binder, 2012). Animal studies indicate that ELS results in a site-specific upregulation of multiple glucocorticoid receptor transcripts, a net increase in receptor mRNA, and enhanced transcriptional regulation of target genes [for example, increased glucocorticoid receptor occupancy at the intronic glucocorticoid response element (GRE) of FK506 binding protein 51 (Fkbp5)] (Bockmühl et al., 2015). In addition, exposure to ELS interferes with the function and innervation of serotonergic and dopaminergic neurons in the prefrontal-limbic system neural circuit (Gos et al., 2006; Jezierski et al., 2007; Kunzler et al., 2015). Multiple sources of evidence support the regulatory roles of the serotonergic system, the dopaminergic system and other neurotransmitter systems in the pathogenesis of depression (Gershon et al., 2007; van der Doelen et al., 2015; Wang et al., 2017; Marrocco et al., 2019; Alameda et al., 2022). Most of the current commonly used antidepressants, especially selective serotonin reuptake inhibitors (SSRIs), serotonin-norepinephrine reuptake inhibitors, and monoamine oxidase inhibitors, primarily target monoamine neurotransmitter function (Harmer et al., 2017). However, there is no drug aimed at depression-like outcomes produced by different types of ELS or reducing susceptibility to depression. Therefore, the mechanism of different types of ELS-induced depression-like behaviors and their interfering factors have received extensive attention in recent years.

Epigenetic modification is considered a promising pathway to counteract the onset of depression by modulating persistent changes in gene expression in response to ELS. Epigenetics refers to potentially heritable but environmentally modifiable changes in gene expression mediated by non-DNA-encoded mechanisms (Sun et al., 2013; Park et al., 2019). These modifications, including DNA methylation, histone modification, and non-coding RNA, may result in the following changes in genetic transcription, synaptic plasticity, and behavior (Tesone-Coelho et al., 2015; Palmisano and Pandey, 2017; Alameda et al., 2022). By nature, epigenetic mechanisms are dynamic and reversible, and they can be used as a new intervention strategy to treat psychiatric disorders (Lesch, 2011). Stress and depression are primarily associated with epigenetic alterations in genes involved in regulating resilience and/or susceptibility to stress, including stress response-related genes (*crf*) and genes involved in neurotransmission (*SLC6A4*) (Park et al., 2019). In recent years, many studies have focused on the role of epigenetics on depression-like outcomes produced by different types of ELS (Ptak and Petronis, 2010; Alameda et al., 2022). Among various epigenetic modifications, cytosine-phosphate-guanine (CpG) methylation has the longest duration (Guo et al., 2011). In both animal and clinical studies, an unfavorable maternal environment causes epigenetic changes in neurons that are often persistent (Oberlander et al., 2008; McGowan et al., 2009; Oh et al., 2013). Some ELS, such as maternal separation, can induce long-term epigenetic changes in gene expression and even persist into adulthood (Bhansali et al., 2007). Maternal effects indirectly regulate gene expression by regulating splicing selectivity, promoter usage, and microRNAs (miRNAs) expression, rather than regulating transcription from proximal promoters (Oh et al., 2013). Under the action of maternal effect, neurotransmitter receptors (corticotropin-releasing factor receptor type 1 (CRF1), dopamine D3 receptor (DRD3), adenosine A2 receptor (ADORA2A), acetylcholine alpha 4 subunit (CHRNA4), gamma-aminobutyric acid A receptor gamma 3 (GABRG3), and GABA_B_ receptor subunit 2 (GABBR2)) are methylated to varying degrees (Oh et al., 2013).

In this article, we aimed to review recent studies on the epigenetic mechanisms of different types of ELS-induced depression-like behavior, especially focusing on the neurotransmitter systems such as the dopaminergic system, the serotonergic system, and the glutamatergic system. We hoped to find therapeutic targets for people who have experienced childhood stress and trauma.

## Serotonergic System

Periodic maternal separation during pre-weaning leads to altered serotonin concentration and serotonergic function in selective brain regions [nucleus accumbens (NAc), hippocampus, and raphe] (Matthews et al., 2001; van Riel et al., 2004; Jahng et al., 2010). Serotonin (5-HT) level can be regulated by multiple factors, such as synthesis, release, and reuptake (Calabrese et al., 2013). At present, 14 different 5-HT receptor subtypes have been identified, and they belong to seven families (termed 5-HT_1_ through 5-HT_7_). Among them, 5-HT_1_ was divided into six subtypes (termed 5-HT_1A_ through 5HT_1F_), and 5-HT_2_ was divided into three subtypes (termed 5-HT_2A_ through 5-HT_2C_) (Fink and Göthert, 2007; Björk et al., 2010). Many studies indicate that the serotonergic system is involved in the pathogenesis and therapy of depression (Hoyer et al., 2002; Stockmeier, 2003). Experiencing social isolation rearing (single cage feeding on post-natal day 21) results in a decrease in 5-HT and its metabolite 5-hydroxyindole-acetic acid (5-HIAA) in the prefrontal cortex of adult rats (Möller et al., 2013).

### 5-HT_1A_R

The serotonin type 1A receptor (5-HT_1A_R) is considered to be an important specific therapeutic target for depression. 5-HT_1A_R and serotonin type 2C receptor (5-HT_2C_R) modulate reward behavior by modulating dopamine release in the NAc (Leventopoulos et al., 2009). Repeated early deprivation leads to reduce reward motivation and a decrease in 5-HT_1A_R binding in the anterior cingulate cortex (ACC), CA1, and dorsal raphe nucleus (DRN) in adulthood (Leventopoulos et al., 2009). Epigenetic mechanisms of anxiety- or depression-like behavioral changes are associated with maternal 5-HT_1A_R deficiency. In the offspring, the immobility behavior of the second-filial generation of male mice was particularly reduced in the forced swimming test. In addition, the behavioral changes in the first- and second-filial generation of female mice were in opposite directions. These suggest that there may be sex differences in epigenetic mechanisms resulting from maternal 5-HT_1A_R deficiency (Mitchell et al., 2016). Immune system dysregulation in 5-HT_1AR_^+/−^ heterozygote and first-filial generation females was associated with immune activation in their offspring and the transmission of somatic anxiety trait. Non-genetic traits of complex psychiatric-like phenotypes were independently transmitted across multiple generations through parallel non-genetic mechanisms. The features of anxiety and hypoactivity were transmitted through somatic mechanisms, while the altered stress-reactivity was transmitted through gamete mechanisms (Mitchell et al., 2016). *In vitro* experiments further found that differentially methylated regions existed in the first-, second-, and third-filial generations of neurons, and 95% of the methylation changes occurred in CpG dinucleotides (among them, hypomethylation accounts for 55% and hypermethylation accounts for 45%), and 87% of differentially methylated sites were unidirectional (Mitchell et al., 2016).

In clinical studies, ELS induced hypermethylation of *5-HT*_*1A*_ *R*, whereas patients with high *5-HT*_*1A*_ *R* methylation from −340 to −149 bp upstream of the transcription start site (TSS) showed a decrease in 5-HT_1A_R expression (Xu et al., 2022), which further reduced 5-HT_1A_R availability (Xu et al., 2011; David and Gardier, 2016). These are consistent with the results of animal studies.

### 5-HT_2_R

Maternal separation induces the development of adult depression and increases *5-HT*_*2C*_ *R* pre-mRNA editing significantly (Bhansali et al., 2007). Fluoxetine administration in adolescence reduced depression-like behaviors and suppressed the increase in the phenotype of *5-HT*_*2C*_ *R* pre-mRNA editing; however, fluoxetine administration in adult mice did not affect either depression-like behaviors or the *5-HT*_*2C*_ *R* pre-mRNA editing phenotype (Bhansali et al., 2007). Mice exposed to ELS showed significantly increased expression of mRNA and protein-encoding the Gαq subunit of G-protein that couples to 5-HT_2A/2C_Rs. The aforementioned results suggest that compensatory changes in Gαq expression occur in mice with persistent changes in *5-HT*_*2C*_ *R* pre-mRNA editing (Bhansali et al., 2007).

*5-HT*_*2A*_ *R* genotype was associated with the methylation of *5-HT*_*2A*_ *R* at CpG-1420 and CpG-1224 in a sample of preschoolers with ELS (Parade et al., 2017). Contextual stress was positively correlated with the methylation of A homozygotes at the CpG-1420 site and negatively correlated with the methylation of G homozygotes at the CpG-1420 site. Depression-like behaviors were negatively correlated with methylation of CpG-1420 and positively correlated with methylation of CpG-1224. Collectively, environmental factors and DNA variation influence the epigenetic process of *5-HT*_*2A*_ *R* (Parade et al., 2017).

### 5-HT_3_R

Serotonin type 3A receptor (5-HT_3A_R) is required for exercise-induced neurogenesis and antidepressant effects, and it modulates cortical interneuron migration and dendritic morphology in pyramidal neurons (Murthy et al., 2014; Kondo et al., 2015; Perroud et al., 2016). In clinical studies, early life trauma interacts with *5-HT*_*3A*_ *R* and brain-derived neurotrophic factor (*Bdnf*) genes to exacerbate the risk for depression (Gatt et al., 2010a; Gatt et al., 2010b). The study found that emotional neglect in children was inversely correlated with methylation levels of CpG1_I (located in the GRE element upstream of *5-HT*_*3A*_ *R*). In addition, a functional *5HT*_*3A*_ *R* single nucleotide polymorphism (SNP) (rs1062613) selectively affects the methylation of a CpG located at 1 bp of the SNP (Perroud et al., 2016). However, the relationship between depression-like outcomes produced by different types of ELS and the epigenetic modification of *5HT*_*3A*_ *R* and its mechanism still needs to be explored.

### SERT

The serotonin transporter is encoded by a single gene, *SERT* (also known as *5-HTT* or *SLC6A4*) (Caspi et al., 2003), located in the presynaptic 5-HT nerve terminal, axons, and cell bodies (Blakely et al., 1998). In the brain, SERT modulates the intensity and duration of serotonergic neurotransmission (Gaspar et al., 2003). Clinical study results show that methylation of the *SLC6A4* promoter is associated with increased susceptibility to depression (Olsson et al., 2010), and higher *SLC6A4* promoter methylation is significantly associated with childhood adversity (Kang et al., 2013). Methylation of *SLC6A4* was positively associated with depression severity in women but not with depression severity and age of onset (Sanwald et al., 2021).

There is a common polymorphic region in the *SERT* gene, the 5-HT transporter-linked polymorphic region (5-HTTLPR), and carriers of the 5-HTTLPR short (S) variant have an increased susceptibility to depression under adversity (Uher and McGuffin, 2010). Interestingly, one study found that sex determined neonatal *SLC6A4* methylation independent of ELS and *5-HTTLPR* genotype, and the methylation was higher in females than males (Dukal et al., 2015). The short allele of the 5-HTTLPR polymorphism and maternal prenatal stress/child maltreatment showed an additive relationship to the reduction of *SLC6A4* mRNA expression *in vivo* (Wankerl et al., 2014). Deletion of the *SERT* led to depression-like behavior, which may be associated with reduced neuronal plasticity (Lira et al., 2003). Female offspring of mice after maternal immune activation have enhanced anhedonia behavior, as manifested by a reduced preference for sucrose (Reisinger et al., 2016). In addition, in the hippocampus, the binding of acH3 and acH4 histones to the *SERT* promoter was increased nearly twofold, suggesting that *SERT* is a specific target for the regulation of epigenetic changes induced by maternal immune activation (Reisinger et al., 2016).

In the prefrontal cortex, *Bdnf* mRNA levels were more sensitive to the changes in *SERT*. *SERT* deficiency significantly reduced *Bdnf* mRNA expression in rat prefrontal cortex during the first week of life, whereas changes in the ventral hippocampus were not seen until the second week (Pezawas et al., 2008; Calabrese et al., 2013). This may be related to the degree of promoter methylation of *Bdnf* exon IV in *SERT* ^−/−^ rats. Further study found that the level of DNA methyltransferase, DNA (cytosine-5)-methyltransferase 1 (*Dnmt1*) was increased in *SERT* ^−/−^ rats, while the level of demethylase growth arrest and DNA-damage-inducible beta (*Gadd45β*) was decreased. In addition, the induction of depression- or anxiety-like behaviors by *SERT* deletion is closely associated with increased DNA methylation of *Bdnf* and decreased availability of transcription factors such as cAMP-response element-binding protein (*Creb*), neuronal PAS domain protein 4 (*Npas4*), and calcium-responsive transcription factor (*Carf*) (Molteni et al., 2010; Calabrese et al., 2013). Furthermore, the study found that serum *SERT* mRNA levels were reduced in individuals exposed to maternal prenatal stress or child maltreatment, but this phenomenon was not observed in stressed adults, suggesting that this change occurs during a sensitive period of early development (Wankerl et al., 2014). In addition, depressive symptoms in second-trimester women were positively associated with methylation within subregions of *SERT* CpG island (Devlin et al., 2010), and the methylation status of the gene promoter was closely related to the volume of the hippocampus, especially in the dentate gyrus, CA2, and CA3 of in the hippocampus (Booij et al., 2015).

### CRF

Changes in 5-HT content regulate *crf* mRNA level in the paraventricular nucleus of the hypothalamus (PVN) (Jørgensen et al., 2002), and SSRIs administration reverses stress-induced *Crf* transcription elevation (Pan et al., 2013). CRF is a 41 amino acid peptide. Hyperactivity of the CRF neuronal system appears to be a pathological hallmark of depression, and CRF is a key mediator of the hypothalamic-pituitary-adrenal (HPA) axis (Hasan and Hasan, 2011). In response to stress, CRF initiates a series of physiological processes and ultimately releases glucocorticoids from the adrenal cortex (Smith and Vale, 2006), and HPA axis hyperactivity returns to normal after antidepressant treatment (Arborelius et al., 1999). It has been found that maternal separation alters CRF expression in brain regions such as the central amygdala (CeA), PVN, and bed nucleus of the stria terminalis (BNST) (Chen et al., 2012). In the female mice which were exposed to impoverished housing, increased risk-taking behavior during a reward-related task (predator-odor risk-taking, PORT) was associated with increased CRF receptor 1 (*Crfr1*) gene expression in the medial prefrontal cortex. Further studies revealed that the levels of the protein marker histone H3 at arginine 2 (H3R2me2s) in the proximal promoter region of the *Crfr1* gene were elevated, whereas these changes were not observed in male mice (Viola et al., 2019). The deposition of histone-modified H3R2me2s results in a stable euchromatin structure that is essentially associated with transcriptional activation (Migliori et al., 2012), which further confirms the phenomenon of elevated *Crfr1* mRNA levels after PORT testing (Schreiber et al., 2017; Viola et al., 2019). In addition, maternal separation and 5-HT genotypes affect the epigenetic modification of the urocortin 1 (*Ucn1*) gene (a member of the CRF peptide family). Studies have found that maternal separation induces methylation at CpG-156 and CpG-49 of the *Ucn1* promoter region in the Edinger–Westphal nuclei, and 5-HTT deficiency induces DNA methylation at CpG-171 in the *Ucn1* promoter region; however, DNA methylation at other CpG sites was not affected (van der Doelen et al., 2017). The interaction of maternal separation and *5-HTT* genotype affects DNA methylation of the *Crf* gene promoter in adult rat CeA, while DNA methylation at specific sites in the *Crf* promoter was related to *Crf* mRNA levels in CeA, and CpG 36 negatively correlated with CeA *Crf* mRNA levels (van der Doelen et al., 2015).

### TPH2

The tryptophan hydroxylase 2 (*TPH2*) gene encodes a rate-limiting enzyme involved in the 5-HT synthesis (Tesoro-Cruz et al., 2021). Clinical studies have shown that the association of *TPH2* methylation changes with ELS only manifests at specific CpG sites. Men with depression who experienced ELS had hypermethylation at the TPH2-5-203 CpG site, whereas depressed women who experienced ELS had hypermethylation at the *TPH2*-10-60 CpG site. These methylations alter the transcription of *TPH2*, further disrupting 5-HT levels, thereby counteracting the effects of antidepressants (Shen et al., 2020).

## Dopaminergic System

Dopaminergic system dysfunction is a pathological hallmark of many neuropsychiatric diseases, such as depression, anxiety, and drug addiction. Stress induces changes in dopamine release or metabolism in the dopaminergic system, especially in the mesolimbic dopaminergic system. The release of dopamine typically varies according to the intensity, duration, and avoidance ability of stress (Baik, 2020). The reduction of dopaminergic function and dopaminergic activity may lead to depression-like behaviors in rodents (Willner et al., 1992; Di Chiara et al., 1999; Jahng et al., 2010). Dopamine receptors are divided into two categories, the dopamine 1 family [divided into dopamine D1 receptor (DRD1) and DRD5 subtypes] and the dopamine 2 family (divided into DRD2, DRD3, and DRD4 subtypes) (Dunlop and Nemeroff, 2007).

### DRD1

It has been reported that maternal separation combined with social isolation stress reduced *Drd1a* mRNA expression, while *Drd2* mRNA expression did not change significantly (Sasagawa et al., 2017). In addition, stress increased methylation of the promoter of the *Drd1a* gene in mice NAc, and 29 of 31 CpG sites (CpG sites 1–5, 8–16, and 18–31) in the *Drd1a* gene were more frequently methylated than normal mice (Table 1) (Sasagawa et al., 2017). It has been found that maternal separation induces an increase in DNA methyltransferase expression in pups and adult rats NAc (Anier et al., 2014; Todkar et al., 2015). According to a recent study, demethylation of histone H3 on lysine 79 (H3K79me2) was a key regulator of transcriptional abnormalities in the adult NAc induced by maternal separation (Kronman et al., 2021). In addition, short-term ELS increased DRD1 expression in the hippocampus and decreased the expression of histone H3 acetylation and dopamine- and cAMP-regulated neuronal phosphoprotein (DARPP-32) but did not change the level of histone H4 acetylation. In contrast, long-term maternal separation upregulated DARPP-32 expression but did not alter DRD1 expression and histone H3/H4 acetylation (Köhler et al., 2019). Furthermore, it was confirmed that the short-term maternal separation induced a decrease in the expression of DARPP-32 was related to the decrease of acetylation of H3 in the promoter region but not to the acetylation of H4 (Köhler et al., 2019).

**TABLE 1:** Summary of different types of ELS-induced epigenetic changes in neurotransmitter system-related genes in animal models mentioned in this review.

No.	Gene name	Animal	Model	Period	Tissue	Neurotransmitter system	Biological function	Reference
1	*Drd1a*	C57BL/6N female mice	Maternal separation coupled with social isolation	3 h daily from PND 1 to PND 14	VTA; NAc	Dopaminergic system	*Drd1a* DNAm↑;	Sasagawa et al. (2017)
2	*Drd2*	Pregnant Wistar rats	Utero glucocorticoids exposure	Gestation day 18 and gestation day 19	NAc	Dopaminergic system	Cell number and dopamine level↓; *Drd2*↑	Rodrigues et al. (2012)
							After morphine administration: *Drd2* DNAm↑	
	*Drd2*	Pregnant adult Sprague–Dawley rats	Maternal deprivation	3 h daily from PND 1 to PND 14	NAc	Dopaminergic system	microRNA-9↓→DRD2↑→stress sensitivity↑	Zhang et al. (2015)
3	*DARPP-32*	C57BL/6 mice	Short-term separation stress; long-term separation stress	Short-term: 3 h daily from PND 14 to PND 16; long-term: 3 h daily from PND 1 to PND 21	HP	Dopaminergic system	Short-term stress: *Drd1*↑, *DARPP-32*↓, H3 acetylation in the *DARPP-32*↓;	Köhler et al. (2019)
							long-term stress: *DARPP-32*↑	
4	*HDAC5*	Wild type and HDAC5^+/−^ offspring	Early social isolation; early social threat	Early social isolation: 30 min daily from PND 14 to PND 21; early social threat: 30 min daily from PND 14 to PND 21	Dorsal striatum	Dopaminergic system	Susceptible to social isolation-induced adverse effects↑	Valzania et al. (2017)
5	*AKAP5*	Male Sprague–Dawley rats	Maternal deprivation	24 h at PND 9	VTA	Dopaminergic system	Transcription of *AKAP5*↓	Shepard et al. (2018)
6	*TH*	Sprague–Dawley rats	Neonatal maternal separation	3 h a day from PND 1 to PND 14	VTA; SN	Dopaminergic system	*TH*↑	Jahng et al. (2010)
7	*5-HT*_*2C*_ *R*	BALB/cJ mice	Maternal separation	3 h a day from PND 2 to PND 15	Forebrain neocortical	Serotoninergic system	*5-HT*_*2C*_ *R* pre-mRNA editing↑	Bhansali et al. (2007)
8	*Crf*	Serotonin transporter knockout rats	Repeated and prolonged maternal separation	180 min daily from PND 2 to PND 14	CeA	Serotoninergic system	*Crf* DNAm↓→escape latency↑	van der Doelen et al. (2015)
9	*SERT*	C57BL/6N mice	Maternal immune activation	First 12.5 days of pregnancy	HP	Serotoninergic system	The binding of acH3 and acH4 histones to *SERT* promoter↑→anhedonia behavior↑	Reisinger et al. (2016)
10	*Grin1*	C57, DBA mice	Maternal separation and early weaning	Maternal separation: 4 h daily from PND 2 to PND 5; 8 h daily from PND 6 to PND 16; PND 17 starts early weaning	mPFC	Glutamatergic system	*Grin1* and *ID3*↓→immobility time↑	Montalvo-Ortiz et al. (2016)
11	*Grin2b*	BALB/c mice	Utero bisphenol A exposure	Gestation day 0 to gestation to 19	HP	Glutamatergic system	*Grin2b* DNAm↑	Kundakovic et al. (2015)

*Note*: Drd1a, dopamine receptor D1; DARPP-32, dopamine- and cAMP-regulated neuronal phosphoprotein; HDAC5, histone acetylation enzyme 5; AKAP5, A-kinase anchoring protein 5; Drd2, dopamine D2 receptor; 5-HT2CR, serotonin type 2C receptor; TH, tyrosine hydroxylase; Crf, corticotropin-releasing factor receptor type 1; SERT, serotonin transporter; Grin1, NMDA R1 receptor; Grin2b, NMDA receptor 2b subunit; ID3, DNA-binding protein inhibitor ID-3; NAc, nucleus accumbens; VTA, ventral tegmental area; HP, hippocampus; SN, substantia nigra; CeA, central amygdala; Crf, corticotropin-releasing factor; 5-TT, serotonin transporter; ELS, early life stress; EWcp, centrally projecting Edinger–Westphal nucleus; mPFC, medial prefrontal cortex; ELS, early life stress; CpG, cytosine–phosphate–guanine; PND, postnatal day; DNAm, DNA methylation. The up arrow indicates the increase in expression or related behavior, the down arrow indicates the decrease in expression or related behavior, and the rightward arrow indicates mediated relationships.

### DRD2

H3K79me2 demethylation and the enzymes that control this demethylation [disruptor of telomeric silencing 1-like (DOT1L) and lysine-specific demethylase 2B (KDM2B)] in D2-medium spiny neurons (MSNs) are critical for maternal separation-induced stress sensitivity (Kronman et al., 2021). In adult mice, knockout of the DOT1L or overexpression of the KDM2B in NAc D2-MSN neurons attenuated maternal separation-induced depression-like behavior, including increased social interaction, increased open field exploration, and decreased despair-like behaviors, whereas the same treatment in Nac D1-MSNs had no changes in depression-like behaviors (Kronman et al., 2021). Increased DOT1L after maternal separation is associated with H3K29me2 deposition at numerous genomic loci. Meanwhile, systemic administration of a small-molecule DOT1L inhibitor reversed maternal separation-induced behavioral deficits (Kronman et al., 2021). This provides strong evidence support for the treatment of maternal separation-induced depression.

One of the closely related and persistent histone modifications in ELS is a histone deacetylase (HDAC)-mediated histone acetylation (Levine et al., 2012; Valzania et al., 2017; Shepard et al., 2018). Histone acetylation, the most reported histone modification in neurological diseases, is dynamically regulated by two distinct types of enzymes, histone acetyltransferases (HATs) and HDACs (Marmorstein and Zhou, 2014; Alameda et al., 2022). HAT transferred acetyl groups to histone tails, leading to chromatin relaxation and subsequent increase in transcription rate; the latter increased chromatin-histone interactions by removing acetyl groups and ultimately decreased transcription rate (Shepard and Nugent, 2020). Studies have shown that HDAC inhibitors exert antidepressant-like effects and alleviate depression-like and addictive behaviors (Covington et al., 2009). Studies have found that spatial and associative memory functions are impaired in histone acetylation enzyme 5 (HDAC5) knockout mice and in early-life stressed mice (Agis-Balboa et al., 2013; Suri et al., 2014; Valzania et al., 2017). In addition, a heterozygous null mutation in HDAC5 increased the time of conditioned place preference in mice that suffered from social isolation in early life but not in socially threatened mice (Valzania et al., 2017). The dorsal striatum may be involved in mediating this effect (Valzania et al., 2017). In brief, individuals are more susceptible to social isolation-induced adverse effects when heterozygous null mutations in HDAC5 are present in the organism.

In another study, it was found that maternal deprivation-induced GABAergic neuroplasticity (not glutamatergic) and aberrant A-kinase anchoring protein (AKAP) signaling could be reversed by injection of HDAC inhibitors in the ventral tegmental area (VTA), and researchers proposed these effects may be related to dopamine neurons in the VTA (Authement et al., 2015; Shepard et al., 2018). Further studies found that maternal deprivation increased HDAC2 expression in VTA dopaminergic neurons and increased BDNF (a biological indicator closely related to the onset of depression) expression in VTA (Shepard et al., 2018). In addition, prenatal stress regulated gene expression in the hippocampus, such as decreased early growth response 1 (*Egr1*) and RAC-alpha serine/threonine-protein kinase (*Akt1*) mRNA expression, which were important for regulating cell proliferation and cell survival (Boulle et al., 2016). On the other hand, maternal deprivation also induced histone hypoacetylation in VTA. In addition, maternal deprivation increased the intrasynaptic AKAP150 level and decreased the protein kinase A (PKA)-RIIβ level, and these effects can be reversed by an HDAC inhibitor (Shepard et al., 2018). Maternal deprivation-induced AKAP150-anchored signaling changes may be closely linked with HDAC2-mediated epigenetic modifications, and these modifications prevent transcription of the *AKAP5* gene (Shepard et al., 2018; Shepard et al., 2020). In recent research, the interaction of PKA and AKAP150 regulated the cellular excitability and intrinsic membrane properties of VTA dopaminergic neurons, and the disruption of the AKAP150-PKA complex increased VTA action potential generation in normal animals, and it decreased in maternal deprivation animals (Shepard et al., 2020). Therefore, by targeting AKAP150 and HDACs to reduce changes in histone acetylation, they can modulate not only neuronal excitability through synaptic transmission but also ion channel activity and postsynaptic ion channel transport (Shepard et al., 2020).

ELS enhances stress sensitivity in adulthood by reducing microRNA-9 expression, which, in turn, upregulates DRD2 expression in the NAc (Zhang et al., 2015). It has been found that chronic unpredictable stress increased *Drd2* mRNA levels within the NAc and decreased microRNA-9 expression, while maternal deprivation synergistically enhanced the effects of chronic unpredictable stress on *Drd2* and microRNA-9 expression. *In vitro* studies have shown that microRNA-9 directly targets the 3′ untranslated region of *Drd2* mRNA and then inhibits DRD2 expression (Zhang et al., 2015).

Exposure to *in utero* glucocorticoids significantly decreased cell number and dopamine levels and significantly upregulated *Drd2* mRNA levels in the NAc of adult rats, while repeated morphine administration downregulated the levels of DRD2 expression while increasing the DNA methylation of the *Drd2* gene. Administration of therapeutic doses of levodopa restored a hypodopaminergic state, normalized DRD2 expression, and resisted morphine-induced methylation of the *Drd2* promoter in the NAc of animals exposed to *in utero* glucocorticoids. In addition, levodopa treatment also promoted the dendritic and synaptic plasticity of the NAc (Rodrigues et al., 2012).

### Other Dopamine-Related Genes

Intrasynaptic dopamine transmission is primarily regulated by dopamine transporter (DAT) uptake of released dopamine, and it is sensitive to changes in DAT density and its function (Wightman and Zimmerman, 1990). A lower density of DAT sites in mice NAc may be the reason why maternal separation animals are more susceptible to stress responses (Brake et al., 2004). In addition, in the same experiment, it was found that *Drd3* mRNA levels were greatly reduced in the NAc of maternal stress model mice (Brake et al., 2004).

Tyrosine hydroxylase (TH) is regarded as a rate-limiting enzyme in catecholamine biosynthesis. It has been found that stress response is associated with TH activity in the central nervous system (Masserano and Weiner, 1983). A study found that restraint stress increased *TH* mRNA level and enhanced the rate-limiting enzyme in dopamine synthesis in the midbrain VTA and substantia nigra (SN). In addition, restraint stress-induced increase in *TH* mRNA expression was significantly attenuated in the VTA and SN of neonatal maternal separation-treated rats (Jahng et al., 2010). However, there are few studies on the mechanism of different types of ELS-induced TH expression changes, and further exploration is needed.

## GABAergic System

There are three subtypes of GABA receptors: GABA_A_ receptors, GABA_B_ receptors, and GABA_C_ receptors. GABA_B_ receptors are metabotropic receptors, and GABA_A_ receptors and GABA_C_ receptors are ionotropic receptors (Bormann, 2000). GABA receptors play a pivotal role in the regulation of psychiatric disorders such as depression, epilepsy, and anxiety. Maternal deprivation-induced GABAergic neuronal plasticity and aberrant AKAP signaling could be reversed by the injection of HDAC inhibitors in the VTA (Authement et al., 2015). Maternal separation altered the levels of 24 miRNAs in the hippocampus. Among them, antidepressant treatment attenuated maternal separation-induced changes in the levels of miR-451, a miRNA that regulates many important genes, including GABAergic (GABA_A_ receptor-associated protein) and cholinergic neurotransmission (muscarinic cholinergic receptors 5) (O’ Connor et al., 2013). In the ventral hippocampus of *SERT* ^−/−^ rats at the third week of life, *SERT* deficiency resulted in a significant decrease in γ2 subunit of GABA_A_ receptor (*GABA*_*A*_ *γ2*) and glutamic acid decarboxylase 1 (*Gad67*) mRNA levels, while vesicular-GABA transporter (*Vgat*) only showed a trend of decreasing but not statistically significant. On the other hand, in the prefrontal cortex, *SERT* deficiency only reduced *GABA*_*A*_ *γ2* mRNA levels (Calabrese et al., 2013). The effects of the prenatal environment on early childhood neurodevelopment are gender-mediated in both animal and human studies (Bale, 2011), in which maternal care and ELS in rats are associated with GABAergic synaptic function and GABA_A_ receptor expression (Diorio and Meaney, 2007). In addition, GABA_B_ receptor subunit 1 (*Gabbr1*) gene expression was reduced by 36% in the hippocampus of female rat pups exposed to prenatal stress (Van den Hove et al., 2013). In clinical studies, DNA methylation of *Gabbr1* was positively associated with anxiety during pregnancy in male neonates (Vangeel et al., 2017). The glutamatergic and GABAergic system was critical for stress response and emotion regulation (Popoli et al., 2011). In the results of RNA sequencing, although the expression of the N-methyl-D-aspartic acid receptor (NMDA) receptor subunit genes NMDA R1 receptor (*Grin1*), *Grin2a*, GABA_B_ receptor 2 (*Gabbr2*), and GABA_A_Rα1 (*Gabra1*) was reduced in CA3 after exposure to acute-swim stress, the expression levels of these genes did not change after acute-swim stress in mice exposed to maternal separation (Marrocco et al., 2017; Marrocco et al., 2019).

## Glutamatergic System

In patients with depression, alterations in the glutamatergic system can lead to alterations in related excitatory neurotransmission, and it plays a vital role in the pathogenesis of neurological disorders (Lener et al., 2017; Alameda et al., 2022). It has been found different types of ELS change oxidative stress and redox balance, including elevated levels of the oxidative stress marker Nox2, which controls glutamate release in the prefrontal cortex (Schiavone et al., 2009; Sorce et al., 2010; Möller et al., 2013). In addition, glutamate can bind to ionotropic receptors [including NMDA receptors, α-amino-3-hydroxy-5-methyl-4-isoxazole-propionic acid receptor (AMPA) receptors, and kainate receptors] and metabotropic receptors (mGluRs) (Groc and Choquet, 2006; Lener et al., 2017).

### NMDA

In clinical studies, ELS decreased NMDA receptor expression and increased glutamate-mediated excitotoxicity, which further induced neuronal loss and ultimately reduced NMDA receptor binding (Underwood et al., 2020). In animal studies, it has been found that exposure to maternal separation at 2–3 weeks postnatally altered synaptic plasticity in susceptible rats and inhibited the effect of antidepressants on CA1, accompanied by a lifetime upregulation of synaptic NMDA receptor 1 (NR1) levels in sensitive rats (Ryan et al., 2009). In the epigenome study of children, methylation at CpG sites in DNA-binding protein inhibitor ID-3 (*ID3*), tubulin polymerization promoting protein (*TPPP*), and *Grin1* were inversely related to depression severity in maltreated children (Weder et al., 2014). Similarly, in maternal separation and early weaning (MSEW)–exposed mice, *TPPP* gene expression in the prefrontal cortex was inversely proportional to dwell time in the open arm and proportional to dwell time in the closed arm. The expression of *Grin1* and *ID3* genes in the medial prefrontal cortex were inversely proportional to the immobility time in the forced swimming test (Montalvo-Ortiz et al., 2016), and these were consistent with previous results.

Bisphenol A (BPA) exposure induced sex difference in methylation of the CREB-binding site (CpG1) and a site adjacent to CpG1 of the NMDA receptor 2b subunit (*Grin2b*) promoter, and BPA also induced hypermethylation of CpG3 in *Grin2b* promoter in males in the mice hippocampus (Kundakovic et al., 2015). Similarly, the effect of ELS on the methylation of the *Grin2b* gene has been demonstrated in clinical studies, and the *Grin2b* regulatory region has a higher degree of methylation in adults who have experienced childhood adversity, especially the changes at the CpG3 site in the *Grin2b* gene (Engdahl et al., 2021). These studies confirm that the methylation of the *Grin2b* gene is very sensitive to ELS, and these findings may increase the understanding of the impact of ELS on neurodevelopment at the molecular level.

### AMPA

Studies have found that exposure to early-life social isolation for 6 weeks in 21-day-old rats led to depression-like behaviors, including anhedonia and increased despair-like behavior (Fone and Porkess, 2008; Haj-Mirzaian et al., 2015; Wang et al., 2017). Further studies found that early-life social isolation increased the levels of di-methylation of histone H3 at lysine9 (H3K9me2) in the rat hippocampus, but not tri-methylation of histone H3 at lysine4 (H3K4me3). H3K9me2 has been reported to lead to impairment of synaptic plasticity and glutamatergic neurotransmission and is regarded as a risk factor for major depressive disorder (Muchimapura et al., 2002; Peter and Akbarian, 2011). Furthermore, early-life social isolation reduced the levels of glutamate receptor subunits (NMDA receptor subunits, NR1, and AMPA receptor subunits, GluR1 and GluR2) in the rat hippocampus, which could be rescued by minocycline (Wang et al., 2017). In contrast, maternal isolation increased NMDA receptor 2 expression in adolescent mice (Wieck et al., 2013).

## Treatment of Different Types of ELS-Induced Epigenetic Changes in the Neurotransmitter Systems

At present, studies have found that the epigenetic effects of fluoxetine, citalopram, and other drugs on the serotonergic pathway further reduce depression-like behaviors (Boulle et al., 2016; Unroe et al., 2021). It has been found that developmental fluoxetine exposure (a selective 5-HT reuptake inhibitor for perinatal depression) increased despair-like behaviors in adult rats (Boulle et al., 2016). Further research found that developmental fluoxetine exposure increased Histone H3 Lys 27 trimethylation (H3K27me3, a repressive histone marker) at *Bdnf* promoter IV in the hippocampus in prenatally stressed female offspring. However, this effect was only observed in female offspring exposed to prenatal stress, suggesting that prenatal stress increases vulnerability to developmental fluoxetine-induced epigenetic reprogramming in the hippocampus (Boulle et al., 2016). In addition, in clinical studies, it was found that exposure to SSRIs during pregnancy not only resulted in neonatal abstinence syndrome but also increased the risk of anxiety and autism spectrum disorder in neonates. This may be due to the polymorphism of the *SLC6A4* promoter affecting gene transcription and ultimately altering SERT function. Similarly, offspring of rodents exposed to SSRIs during pregnancy exhibited depression-like and anxiety-like behaviors and increased expression of histone deacetylase 4 (*Hdac4*) in the hippocampus, accompanied by increased H3 and H4 acetylation at the *Hdac4* promoter. In addition, overexpression of Hdac4 in the hippocampus reversed the depression-like behaviors induced by SSRI exposure during pregnancy. These studies provide a potential therapeutic target for depression-like behaviors induced by exposure to SSRIs during pregnancy. On the other hand, these studies could rationalize antidepressant use during pregnancy to avoid adverse effects on offspring (Sarkar et al., 2014; Glover and Clinton, 2016).

Early maternal separation increases susceptibility to depression in chronic mild stressed rats (Choi et al., 2021). N-3 PUFA ameliorated HPA axis dysregulation and BDNF-serotonergic pathway, decreased hippocampal miRNA-218 and miRNA-132 expression, and increased miRNA-155 expression; thus, it had a therapeutic effect on depression induced by maternal separation in childhood combined with chronic mild stress in adulthood (Kim et al., 2020; Choi et al., 2021). Furthermore, quetiapine treatment reversed depression-like behavior and reduced DNA methyltransferase activity induced by maternal deprivation (Ignácio et al., 2017). LPM570065, a 5-HT/NE/DA triple reuptake inhibitor with a high binding affinity for SERT, norepinephrine transporter (NET) and DAT, has been shown to be effective in major depressive disorder treatment in clinical studies. A study found that LPM570065 significantly ameliorated maternal separation combined with social defeat-induced depression susceptibility in adulthood, and this effect was shown to be mediated by reversing *Oxtr* methylation and regulating the expression of DNMT1 and DNMT3a in the hippocampus (Meng et al., 2022). In addition, electroconvulsive shock therapy and ketamine treatment shared 43 miRNA targets after maternal separation, seven of which were found to reverse stress-induced changes after treatment. This also suggests that the antidepressant effects of electroconvulsive shock therapy and ketamine are mediated through a common pathway that converges on the same miRNAs. Whether these miRNAs are worthwhile as therapeutic targets still requires more research (O’Connor et al., 2013).

## Conclusion

**FIGURE 1:**
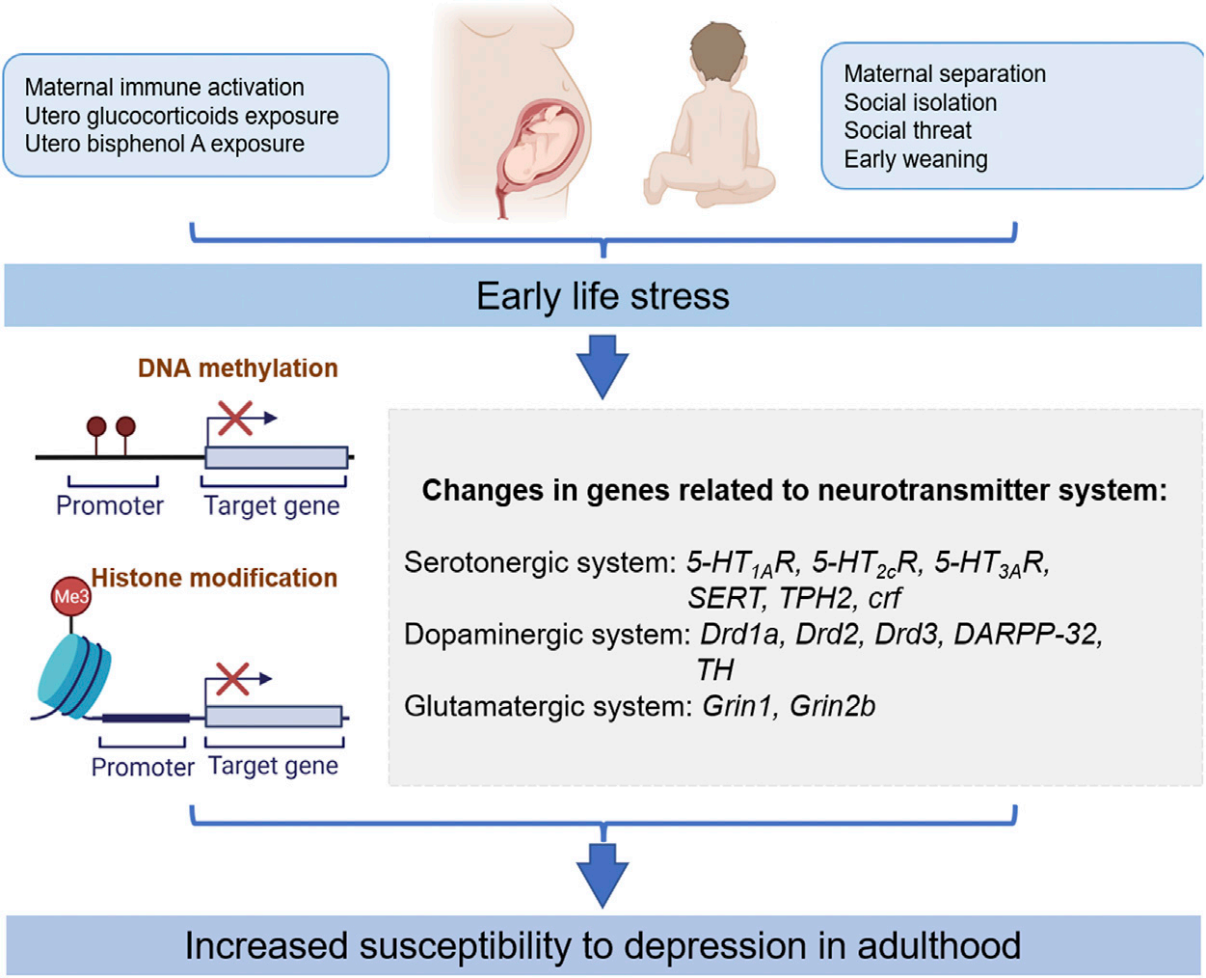
Different types of ELS-induced epigenetic modifications contribute to susceptibility to depression in adults. Drd1a, dopamine receptor D1; Drd2, dopamine D2 receptor; Drd3, dopamine D3 receptor; DARPP-32, dopamine- and cAMP-regulated neuronal phosphoprotein; TH, tyrosine hydroxylase; 5-HT_1A_R, serotonin type 1A receptor; 5-HT_2C_R, serotonin type 2C receptor; 5-HT_3A_R, serotonin type 3A receptor; SERT, serotonin transporter; TPH2, tryptophan hydroxylase 2; Grin1, NMDA R1 receptor; Grin2b, NMDA receptor 2b subunit; CRF, corticotropin-releasing factor.

In this article, we reviewed the important role of epigenetics in the treatment of depression-like outcomes produced by different types of ELS, particularly the evidence for the neurotransmitter system. We believe that there is a strong link between ELS, epigenetic modifications, neurotransmitters system, and depression. Current studies have demonstrated that epigenetic changes (DNA methylation and acetylation) in the dopaminergic system, serotoninergic system, GABAergic system, and glutamatergic system have a regulatory effect on depression-like outcomes produced by different types of ELS (Figure 1). On the other hand, the neuronal function can be improved by modulating environmental factors, and different types of ELS-induced gene defects can also be normalized by pharmacological intervention. These studies provide direct evidence for the epigenetic mechanism of depression-like outcomes produced by different types of ELS, provide new targets for the treatment of depression, and provide a theoretical basis for the development of more effective drugs in the future.
